# Characterization and management of serpentine supravenous reaction: A nine-patient case series

**DOI:** 10.1016/j.jdcr.2026.07.009

**Published:** 2026-07-09

**Authors:** Katie Lu, Gabrielle Arkema, Jarish N. Cohen, Rony A. Francois, Julie Y. Ramseier, Allison S. Dobry

**Affiliations:** a School of Medicine, University of California, San Francisco, California; b Department of Dermatology, University of California, San Francisco, California; c Helen Diller Family Comprehensive Cancer Center, University of California, San Francisco, California

**Keywords:** chemotherapy, drug-induced pigmentation, hyperpigmentation

## Introduction

Serpentine supravenous hyperpigmentation is the term traditionally used to describe hyperpigmentation overlying the superficial venous network at sites of intravenous chemotherapy infusion.^1^ For the purposes of this report, we refer to this entity as serpentine supravenous reaction (SSR), and encourage use of this term to reflect its broader clinical and histopathologic spectrum of findings beyond hyperpigmentation alone. SSR has been described most often with antimetabolites, (in particular, 5-fluorouracil), vinca alkaloids (eg, vincristine, vinorelbine), and taxanes (eg, paclitaxel, docetaxel).^2^ The true incidence remains unclear, as reports are limited to individual cases and small series. SSR typically involves the forearm or hand where the peripheral infusion is administered, but can also occur close to infusion sites on the back, thigh, hip, and popliteal fossa.^3^ This reaction generally appears within 2 weeks of infusion but may occur later.^4^ Given the paucity of reports, the clinical and histopathologic spectrum of SSR remains poorly defined, and no standardized grading or management approach currently exists.^5^ Here, we present a nine-person case series of SSR illustrating variability in causative agents, histopathologic features, and disease course, with the goal of improving recognition and management by both oncologists and dermatologists.

## Methods

We conducted a retrospective chart review of patients diagnosed with SSR who were evaluated in a supportive dermato-oncology clinic at a single academic institution between October 2022 and October 2025. Cases were identified through oncology referrals in which the clinical pattern of venous linear hyperpigmentation or erythematous change following chemotherapy infusion was documented. For each patient, data were abstracted on demographics, underlying malignancy, chemotherapeutic agents, time to onset of rash, clinical morphology, histopathology, treatment, and outcomes.

## Results

Nine patients (8 female, 1 male) developed SSR following chemotherapy infusion. The mean age was 54.7 (standard deviation 14.0). Seven patients had breast cancer, 1 had metastatic prostate cancer, and 1 had metastatic adenoid cystic carcinoma. The chemotherapeutic agents implicated were docetaxel (*n* = 7), paclitaxel (*n* = 1), and vinorelbine (*n* = 1).

Cutaneous changes appeared early in the treatment course and were localized to the infused extremity in a venous distribution, most often involving the forearms and antecubital fossae.

Onset occurred an average of 7.0 days (standard deviation 3.8 days) after peripheral chemotherapy infusion, and several patients developed additional or contralateral lesions with subsequent cycles.

Clinical morphology ranged from asymptomatic linear or ovoid hyperpigmented patches (Fig 1) to edematous erythematous plaques with vesiculation (Fig 2). Four patients exhibited vesiculobullous change (tense bullae or clustered vesicles) near the infusion site (Fig 3). Pruritus and tenderness were consistently reported in patients with inflammatory morphologies (ie erythema, bullae). One patient also experienced widespread urticaria several days after onset of SSR, which was deemed unrelated to taxane hypersensitivity.

**Fig 1:**
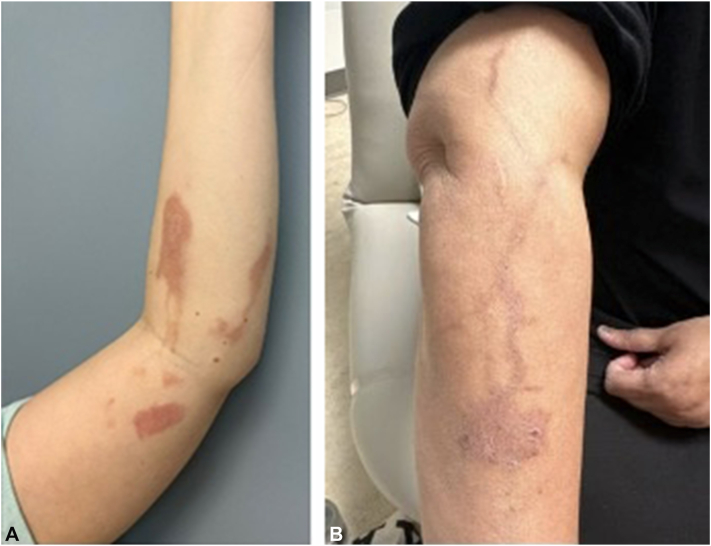
Erythematous serpentine supravenous reaction. **A,** Dull red patches overlying the venous network on the left arm, 3 weeks after peripheral docetaxel infusion into the left volar forearm and 6 weeks after peripheral docetaxel infusion into the left medial forearm. **B,** Supravenous pink to hyperpigmented scaly patch 4 weeks after docetaxel infusion into the right dorsal forearm.

**Fig 2:**
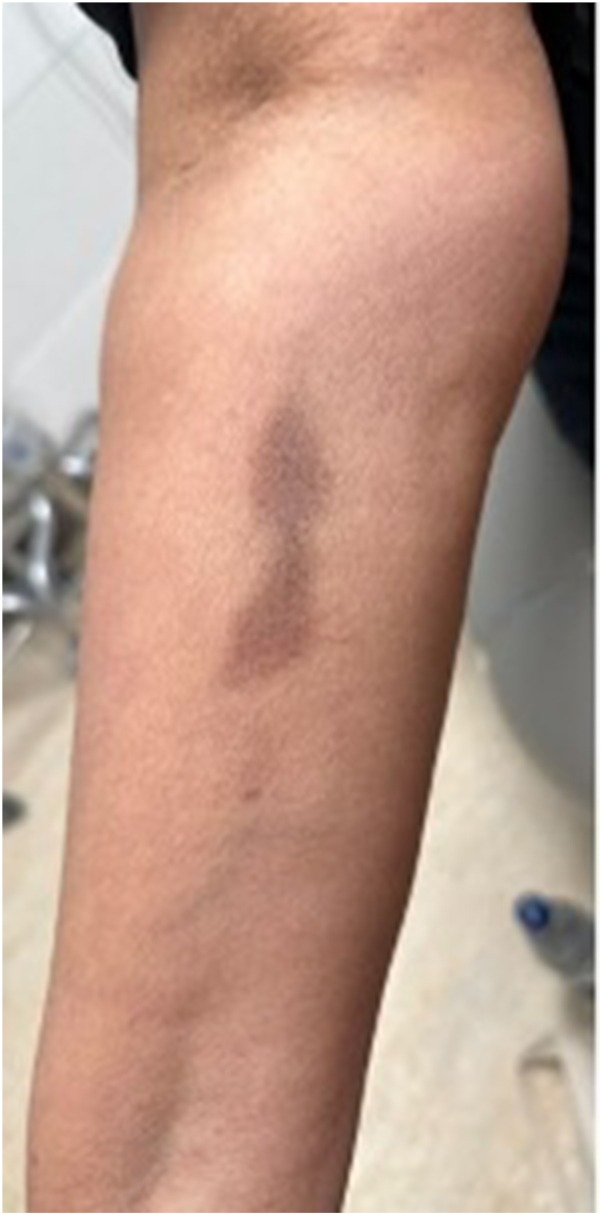
Hyperpigmented serpentine supravenous reaction. Hyperpigmented patch on the right volar forearm, 6 weeks after peripheral docetaxel infusion.

**Fig 3:**
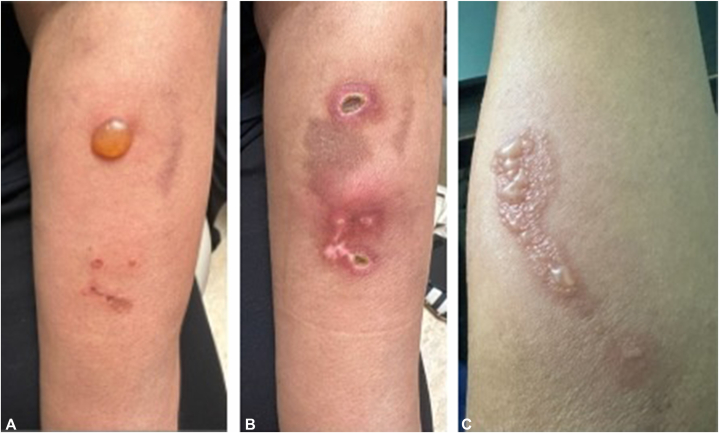
Vesiculobullous serpentine supravenous reaction and sequela. **A,** Tense vesicles and bullae with surrounding erythema 3 days after peripheral docetaxel infusion. **B,** Shallow ulcers with eschars and erythematous borders at sites of prior bullae, 4 weeks after peripheral docetaxel infusion. **C,** Edematous, erythematous plaque with overlying vesicles, 7 days after peripheral docetaxel infusion.

Two patients in our series underwent skin biopsy. Histopathologic findings included atrophic vacuolar interface dermatitis in case 1 and subepidermal vesicular dermatitis with numerous eosinophils in case 4 (Fig 4). Direct immunofluorescence analysis and bullous pemphigoid serologies were negative for the latter patient. In all cases, the overall clinical impressions favored SSR as opposed to extravasation reaction or fixed erythrodysesthesia. However, the histopathologic features seen here are not exclusive to SSR and similar findings may be observed in other entities, including fixed erythrodysesthesia and bullous dermatoses.

**Fig 4:**
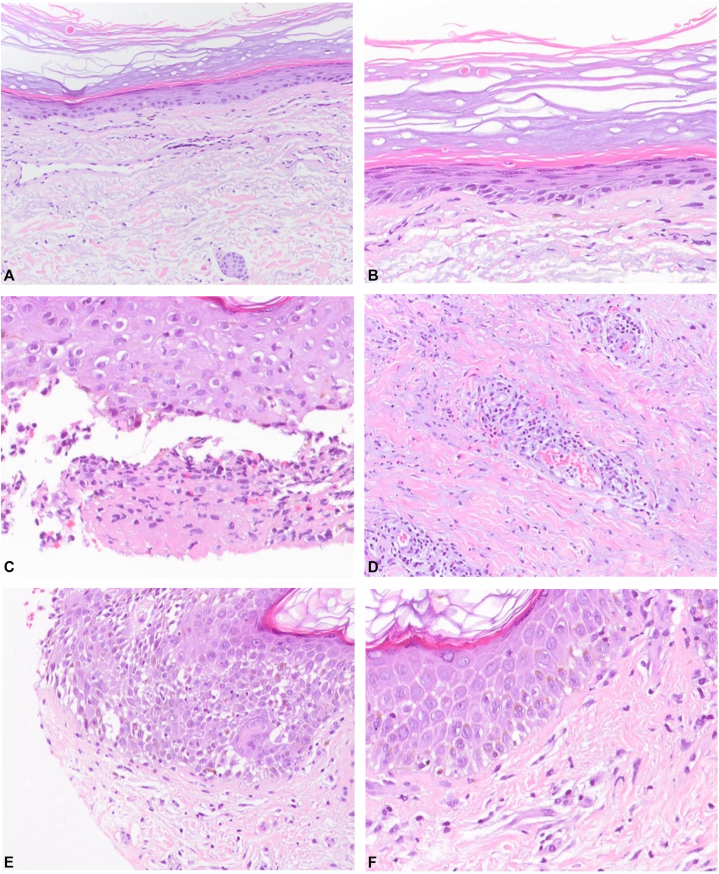
Histopathologic findings from cases 1 **(A** and **B)** and 4 **(C-F)**. Case 1: **(A)** A paucicellular atrophic vacuolar interface dermatitis is present (H&E, 200×). **B,** There is epidermal atrophy with loss of the normal rete ridge pattern and focal homogenization of the superficial papillary dermis (H&E, 400×). Case 4: **(C)** A mixed pattern inflammatory reaction is present, consisting of a subepidermal vesicular dermatitis with subjacent eosinophils (H&E, 400×); **(D)** perivascular and interstitial lymphocytes, eosinophils, and fibrin (H&E, 400×); **(E)** multifocal spongiosis (H&E, 200×); **(F)** occasional necrotic keratinocyes, and rare melanophages (H&E, 400×).

Management consisted primarily of topical therapy. High- or mid-potency topical corticosteroids were used in most cases. Bullae, when present, were lanced and drained. One patient received a short course of systemic prednisone for concurrent urticaria. Another patient pre-treated the subsequent infusion sites with topical triamcinolone and did not develop recurrent bullae, though local hyperpigmentation still occurred. Depigmenting agents (eg, hydroquinone 4% cream, OTC kojic acid cream, vitamin C cream) were prescribed to 2 patients with unclear benefit. Other approaches, including post-docetaxel venous washing and taxane substitution, were recommended but not ultimately employed.

Cases with acute inflammatory vesiculobullous and edematous components resolved within approximately 3 weeks after onset. However, residual dyspigmentation persisted in most patients at last follow-up, though partial fading was noted in 5 patients. Atrophic scars occasionally formed at sites of prior bullae.

## Discussion

Serpentine supravenous hyperpigmentation has traditionally been described as linear or reticular hyperpigmentation following the superficial venous network at sites of chemotherapy infusion. However, reports vary widely with regard to implicated agents, severity, histopathologic findings, and clinical course, and the full spectrum of this entity remains poorly characterized. In the present cohort, we refer to this entity as serpentine supravenous reaction (SSR), and propose this term to reflect its broader clinical and histopathologic features.

In our case series, 78% (7/9) of patients received docetaxel (2 in combination with carboplatin and 3 in combination with cyclophosphamide), 11% (1/9) received adjuvant paclitaxel with trastuzumab, and 11% (1/9) received vinorelbine with cisplatin (Table I). These findings are consistent with prior reports implicating taxanes as a common causative agent, while also broadening the spectrum to include combination regimens and non-taxane agents such as vinorelbine that were not previously well-documented.^6^ Our findings also suggest that onset can be rapid, developing as early as 1 day after infusion.

**Table I:** Clinical characteristics and treatments of patients with SSR

Patientnumber	Age/sex	Cancertype	Infusion drug	Site	Rash onset after infusion	Morphology	Biopsy features	Treatment	Evolution
1	70F	Breast cancer	Paclitaxel and Trastuzumab	Bilateral forearms	6 d	Erythematous supravenous patch and hyperpigmented to pink desquamative ovoid patch	Atrophic vacuolar interface dermatitis	- Clobetasol 0.05% ointment BID, - Tacrolimus 0.1% ointment BID	Very subtle hyperpigmentation noted at 1 y follow up
2	57F	Breast cancer	Docetaxel and Carboplatin	Bilateral forearms	3 d	Tense vesicles and bullae with amber-colored fluid on erythematous bases. Supravenous dull red and hyperpigmented patches at prior infusion sites.	No biopsy	- Bullae lanced - Triamcinolone 0.1% ointment BID - Santyl for eschar debridement. - Prophylactic triamcinolone to forearms prior to further treatment cycles. - For persistent dyspigmentation, compounded hydroquinone 6%/kojic acid 6%/tretinoin 0.025%/hydrocortisone 2.5% cream QHS	Scars at sites of prior bullae, hyperpigmented patches persistent at 10 mo follow up
3	77M	Prostate cancer	Docetaxel	Left antecubital fossa	14 d	Hyperpigmented patches overlying prior infusion sites	No biopsy	- Triamcinolone 0.1% ointment BID	No follow up available
4	61F	Breast cancer	Docetaxel	Right antecubital fossa and volar forearm	7 d	Hyperpigmented patches at prior infusions sites and erythematous, edematous supravenous plaque with overlying tense bullae at most recent infusion site	Mixed pattern subepidermal vesicular dermatitis with eosinophils, spongiosis, perivascular lymphocytes and eosinophils, with necrotic keratinocytes	- Prednisone 60 mg with taper - Hydroxyzine QHS - Triamcinolone 0.1% cream BID - Bullae lanced	Hyperpigmented patches and pink atrophic patches at sites of prior bullae at 2 wk follow up
5	37F	Breast cancer	Docetaxel and Cyclophosphamide	Left forearm	Unknown	Linear supravenous hyperpigmentation without erythema	No biopsy	- OTC vitamin C cream - Hydroquinone 4% cream	Hyperpigmentation faded, but not fully resolved at 1 y follow up
6	45F	Breast cancer	Docetaxel and Carboplatin	Right forearm	7 d	Erythematous patches in a supravenous distribution with large overlying serous vesicles and bullae. Supravenous hyperpigmented patches at prior infusion sites.	No biopsy	- Clobetasol 0.05% BID - Bullae lanced	Subtle hyper- and hypopigmented supravenous patch noted 6 wk after infusion
7	41F	Breast cancer	Docetaxel and Cyclophosphamide	Bilateral forearms	11 d	Erythematous thin edematous supravenous plaques with few overlying collarettes of scale	No biopsy	- Triamcinolone 0.1% ointment BID	Subtle faint pink patches noted 2 mo after initial rash
8	42F	Adenoid carcinoma	Vinorelbine and cisplatin	Right dorsal hand	1 d	Serpentine, red-purple papules with overlying tense bullae on the right dorsal hand	No biopsy	- Clobetasol 0.05% cream BID - Bullae lanced - PICC placed for future infusions	Subtle hypopigmentation noted at 6 wk follow up visit
9	62F	Breast cancer	Docetaxel and Cyclophosphamide	Left forearm	7 d	Erythematous longitudinal supravenous patch	No biopsy	- Mometasone 0.1% cream BID	Hyperpigmentation present 2 wk after initial presentation

The severity and clinical course of SSR varied considerably. Seven patients (78%) exhibited an inflammatory reaction characterized by erythema, pain, pruritus, or blistering (Table II). Among these, 4 patients (44%) developed vesiculobullous changes, and 1 patient (11%) developed desquamative erythematous plaques. All inflammatory reactions resolved before evolving into residual hyperpigmentation or atrophic scars. At present, there is no standardized grading scheme for serpentine supravenous hyperpigmentation, and recognition of its full clinical range is crucial to avoid confusion with extravasation injury, fixed erythrodysesthesia, or hypersensitivity reactions.

**Table II:** Clinical features of patients with SSR

Clinical features	Erythema	Pruritus/burning	Bullae	Hyperpigmentation
Patient 1	X	X		X
Patient 2	X	X	X	X
Patient 3				X
Patient 4	X	X	X	X
Patient 5				X
Patient 6	X	X	X	X
Patient 7	X	X		
Patient 8	X	X	X	
Patient 9	X	X		X

Histopathologic analysis, performed in 2 patients (22%), both demonstrated evidence of an interface dermatitis, either as the predominant pattern, or as part of a mixed inflammatory pattern. While not specific, the histopathologic findings support the diagnosis of SSR in the clinical context of a recent infusion of chemotherapy in the affected extremity. Case 1 demonstrated atrophic vacuolar interface dermatitis, favoring SSR over fixed erythrodysesthesia. The other case consisted of a mixed inflammatory reaction pattern, including a subepidermal vesicular dermatitis with eosinophils, a spongiotic dermatitis, and evidence of a vacuolar interface dermatitis. These findings, coupled with the negative direct immunofluorescence, favored SSR over a delayed vesicant docetaxel extravasation reaction or bullous pemphigoid. However, these microscopic features are not specific and can be seen in other conditions such as erythrodysesthesia, bullous pemphigoid, or hypersensitivity reactions. Furthermore, the variability in histopathologic findings may reflect differences in biopsy timing relative to eruption evolution. This notion is consistent with our recent systematic review, in which the majority of cases with available histopathology had evidence of an interface dermatitis reaction pattern, while other inflammatory patterns, pauci-inflammatory reactions, and specimens without inflammation have also been reported.^6^ Further investigation is needed to determine whether any histopathologic features are unique or predictive of SSR.

Treatment in our series was primarily topical, consisting of high- or mid-potency corticosteroids, draining of bullae when present, and short courses of systemic corticosteroids in selected cases. In general, management of SSR is supportive and directed by the phase of reaction. For acute inflammatory reactions, topical or short-term systemic corticosteroids and cold compresses may help reduce inflammation and discomfort. For residual hyperpigmentation, depigmenting agents have been used with variable success. Preventive strategies, such as post-infusion venous flushing, switching or dose-reducing cytotoxic agents, or prophylactic topical steroid application, have been proposed but not systematically evaluated.^6^

Based on the clinical and histopathologic heterogeneity observed, we propose the term *serpentine supravenous reaction* to replace *serpentine supravenous hyperpigmentation*. This terminology better encompasses the multi-stage cutaneous evolution (including erythematous, edematous, vesicular, and pigmentary phases) and the observed histopathologic findings, rather than implying a purely pigmentary process. Broader recognition of SSR may facilitate more accurate diagnosis, prevent unnecessary work-up for extravasation injury, and expand management strategies.

Future studies are needed to delineate the pathophysiologic mechanisms, define severity grading, and establish treatment algorithms for this under-recognized toxicity. Improved reporting of onset timing will help to define diagnostic criteria and inform supportive care strategies for patients receiving chemotherapy.

## Conflicts of interest

None disclosed.
